# Gastrointestinal and liver adverse effects of anti-tumoral immune therapy: from recognition to treatment 

**Published:** 2021

**Authors:** Simcha Weissman, Saad Saleem, David Aldulaimi

**Affiliations:** 1 *Department of Medicine, Touro College of Osteopathic Medicine, Middletown, NY, USA *; 2 *Department of Medicine, Mercy Saint Vincent Medical Center, Toledo, OH, USA *; 3 *Department of Gastroenterology, South Warwickshire Foundation Trust, Warwick, UK *

**Keywords:** Anti-tumoral immune therapy, Treatment, Gastrointestinal and liver diseases

## Abstract

Anti-tumoral immune therapy consists of monoclonal antibodies that target intra-cellular immune checkpoints—which under normal circumstances, act as regulators of T-cell immunity. By serving as inhibitors of cellular checkpoints, monoclonal antibodies stimulate the immune system thus augmenting the body’s response against cancer. These immune-enhancers or stimulators have revolutionized the treatment of malignancy as they continue to show improvement in the overall survival of cancer patients. Currently, in the United States, six immune checkpoint inhibitors are approved for the treatment of a variety of solid tumors (1). As these checkpoint inhibitors are relatively new, only a scant amount of literature is available regarding both their adverse effects and management thereof. In addition, as newer antibodies are being developed, and expected to be enlisted among the armamentarium of cancer chemotherapeutic agents—the need to understand their toxicity and adverse effects is of paramount importance. Herein, we review some of the gastrointestinal and liver sequelea secondary to the usage of immunotherapeutic checkpoint inhibitor agents in cancer chemotherapy, as well as present the diagnosis and recommended treatment strategies for their adverse effects.

## Checkpoints and inhibitors

 The primary intra-cellular checkpoint proteins that play a major role in cancer pathogenesis and division are the cytotoxic T-lymphocyte-associated antigen 4 (CTLA-4), and programmed cell death-1 (PD-1) checkpoints. These protein checkpoints are located on the T-cells. The physiological role of CTLA-4, PD-1, and their ligands is to restrict immune reaction—preventing tissue damage. Cancer cells use these mechanisms to overcome host defense barriers. Inhibitors or antibodies directed against CTLA-4 and PD-1 stimulate the immune system, which in turn augments the body’s immune response—leading to an accelerated inflammatory response capable of causing tissue damage. Ipilimumab, a CTLA4 protein inhibitor, was the first immune checkpoint inhibitor approved in the United States in 2011 (1, 2). Pembrolizumab and nivolumab act by blocking PD-1 and have also been approved for the treatment of numerous malignancies. 

Since the approval of ipilimumab, pembrolizumab and nivolumab, their efficacy has been confirmed in standard clinical practice. In general, these drugs are less toxic than standard chemotherapy. Their toxicity is different from standard chemotherapy. It is associated with immune-related adverse events— related to their innate ability to enhance the immune system. Although many of the immune check inhibitor side effects are rare, they be fatal (3). 

## Gastrointestinal toxicity

Diarrhea is the most frequently reported advrese effect in patients receiving immune checkpoint inhibitor therapy. The incidence of diarrhea is higher among patients taking combinations of immunotherapy, such as combined anti-CTLA-4/anti-PD-1 therapy (44%), as opposed to those receiving anti-CTLA4 (23–33%) or anti-PD-1 (≤19%) therapy alone (4). Interestingly, mono-therapy with anti-CTLA-4 antibodies is associated with a higher incidence of diarrhea compared to anti-PD-1 therapy. In a trial with ipilimumab in metastatic melanoma patients, the incidence of any grade of diarrhea was reported to be 30 percent, whereas, severe diarrhea (grade 3 or 4) was reported in less than 10 percent of patients (5). Of note, the incidence of diarrhea is dose-dependent. In a phase II dose study, severe diarrhea was reported to be 10 percent at 10 mg/kg dose versus 1 percent in 3 mg/kg dose (6). While the mechanism for immune checkpoint inhibitor mediated diarrhea remains unexplained; it may be due to disruption of the structure and function of gut mucosa secondary to uncontrolled inflammation—thus leading to malabsorption and diarrhea. As checkpoint inhibitors associated diarrhea is a diagnosis of exclusion, it is necessary to rule out other causes of diarrhea including Clostridium difficile and other bacterial/viral infections. Another potentially severe condition that may arise from immune checkpoint inhibitor therapy is colitis. Colitis or enterocolitis is characterised by the presence of diarrhea, fever, and abdominal pain with/or without bloody stools. Although more severe, colitis is a less common sequela of immune checkpoint inhibitor therapy compared to diarrhea. In clinical trials of ipilimumab, the incidence of colitis was reported to be less than five percent (5,6). However, ipilimumab has can induce an extensive and severe form of inflammatory bowel disease (7). Anti-PD-1 antibodies such as nivolumab and pembrolizumab have been more often associated with various forms of enterocolitis (8,9). Although it occurs in less then 1% of cases bowel perforation has also been reported as a consequence of ipilimumab therapy (10). This represents the most severe and life threatening condition associated with immune checkpoint inhibitor therapy. 

## Diagnosis and recognition of gastrointestinal sequelae

As usual, colonoscopy is the most accurate way of diagnosing and evaluating the extent of colitis in a patient complaining of abdominal pain days to weeks after undergoing immune checkpoint therapy. In all cases, biopsy should be performed—as colonic mucosa may appear to be normal, but display significant changes on histology. In addition, immunohistochemical staining should be performed to rule out cytomegalovirus infection (4). Notably, there are two types of anti-CTLA-4 associated colitis reported in the literature. One is a diffuse colitis characterized by mesenteric vessel engorgement, while the other is a segmental colitis with moderate wall thickening and associated pericolonic fat stranding in the segment of preexisting diverticulosis (2). Interestingly, histology differs between anti-CTLA-4 and anti-PD-1 induced colitis. Anti-CTLA-4 therapy induced colitis displays acute inflammation that includes: neutrophilic inflammation with intraepithelial lymphocytes, and crypt epithelial cell apoptosis on histology. Whereas, anti-PD-1-related colitis typically follows one of two patterns: active colitis with apoptosis (active inflammation, neutrophilic crypt micro-abscesses, increased crypt epithelial cell apoptosis) or lymphocytic colitis (increased intraepithelial lymphocytes in surface epithelium and expansion of the lamina propria (4). 

## Managing gastrointestinal sequelae

Before starting treatment, physicians should educate patients regarding the gastrointestinal adverse effects of immune therapy. Patients should be instructed to increase oral hydration if diarrhea develops. Moreover, they should be told to seek medical attention if diarrhea persists, (for more than three days), becomes more severe, or they start to feel dizzy, lightheaded, and/or fatigued. 

Management of diarrhea is based on diarrhea grading. Grade 1 (three or fewer stools per day) can be managed conservatively with proper oral hydration and as an outpatient. Anti-motility agents such as loperamide or oral diphenoxylate atropine sulfate can be added after excluding infectious causes of diarrhea. In grade 1 diarrhea, immunotherapy should be continued unless diarrhea advances. Grade 2 diarrhea involves four to six stools per day above baseline. Immunotherapy should be held temporarily. Patients now require a blood and stool workup that includes CBC, CMP, CRP, ESR, stool calprotectin, and lactoferrin. Notably, abdominal imaging and endoscopy are optional, while the continuation of intravenous (IV) hydration and monitoring of the patient is a must. Grade 3 (diarrhea frequency ≥ 7×/day) and Grade 4 (life threating diarrhea) require a complete blood and stool work up to exclude infectious cause of diarrhea. In Grade 3, temporarily hold the immune therapy, and if diarrhea improves it can be restarted. In Grade 4, permanently discontinue the immune therapy. A gastroenterologist should be consulted in all patients with grade 2 or higher diarrhea, while excluding infection. Patients with grade 2 or higher should be started on high dose prednisone 1 mg/kg/day (4). For patients with an enterocolitis, prednisone 1 mg/kg/day can be increased to 2 mg/kg/hr if no improvement occurs after 48 hours of treatment(4). Once diarrhea improves, steroids should be tapered over 4-6 weeks. Once the steroids are tapered to ≤10 mg/day and patient remains symptom-free, the immune therapy can be resumed. If diarrhea persists on intravenous steroids after three days, the patient should be started on infliximab 5 mg/kg once every two weeks (11-14). Mycophenolate or vedolizumab can be an alternative for cases refractory to infliximab (15,16). A two patient case series suggested the efficacy of fecal microbiota transplantation in colitis patients refractory to standard treatment (17). Budesonide was tried as a prophylactic treatment in a double-blind study; although it has not shown any benefits in preventing diarrhea (18).

## Hepatotoxicity

Anti-CTLA-4 and PD-1 therapy are associated with an asymptomatic elevation of the hepatic enzymes; aspartate aminotransferase (AST) and alanine aminotransferase (ALT). Although liver-related toxicity can occur at any time during treatment, most commonly it occurs 8-12 weeks after treatment initiation (19). As checkpoint inhibitors are relatively new, little data is available addressing the prevalence of liver-related toxicity. In fact, the incidence of AST and ALT elevation with CTLA-4 blockade varied in different clinical trials and observational studies, but was always less than 10 percent (6, 20, 21). In a phase III trial of ipilimumab, the rate of AST/ALT elevation was approximately one to two percent with no reported cases of grade 3 and 4 hepatotoxicity (5). Grade 3 hepatotoxicity occurred in 20 percent with the ipilimumab 3 mg/kg and nivolumab 1 mg/kg combination, and less than 5 percent of patients on a combination of the lower dose of ipilimumab 1 mg/kg and the higher dose of nivolumab 3 mg/kg (22).

In a study examining anti-PD-1 antibodies, the incidence of AST/ALT elevation was less than 5 percent, and grade 3 and 4 toxicity was rare (23-24). The rate of hepatitis may be both more frequent and severe in a combination with chemotherapy agents such as dacarbazine and vemurafenib (25).

## Diagnosis and recognition of hepatotoxic sequelae

A complete medical history including any exposures to alcohol and medications is vital to the evaluation of a patient with abnormal liver enzymes. Imaging is not indicated as specific radiographical findings are typically not seen. Computed tomography (CT) scan may show non-specific findings including hepatomegaly, peri-portal edema, or peri-portal lymphadenopathy (26). Although biopsy is not indicated, it can be performed in complicated cases and may show severe panlobular hepatitis with prominent perivenular infiltrate with endothelialitis (26). Hepatic function should be monitored before initiation and repeated with each dose of ipilimumab, as checkpoint inhibitor induced hepatitis is typically detected on routine liver function tests. (27). Immune checkpoint inhibitor induced hepatitis is a diagnosis of exclusion and thus anyone suspicious for the etiology should have complete blood work up to rule out other causes of hepatitis—such as hepatitis A, B, C, D and E, autoimmune hepatitis, and other drug associated with hepatitis. 

## Managing hepatotoxic sequelae

 After establishing the diagnosis of immune therapy related hepatitis, the patient should be started on corticosteroids and referred to a gastroenterologist (28). In grade 1 hepatitis (AST or ALT 1.25-3 times the upper limit of normal (ULN); or total bilirubin 1.25-2 times the ULN) one should continue to monitor liver function tests (LFT’s ) weekly, unless the patient is stable. In grade 2 hepatitis (AST or ALT 3-5 times the UNL, or total bilirubin 2-3 times the ULN) one should rule out other causes of hepatitis—which include; viral, autoimmune hepatitis, biliary tract obstruction, and metastasis—while checkpoint inhibitor therapy should be held. The patient should be started on prednisone 0.5-1 mg/kg/day with a four-week taper and then resume therapy when corticosteroids are tapered to 10 mg/day. LFT’s should be monitored twice per week. In grade 3 or higher hepatic toxicity (AST or ALT 5-10 times the ULN, or total bilirubin 3-10 times the ULN) the patient should permanently discontinue immune checkpoint therapy and start prednisone 1–2 mg/kg/day. If liver enzymes improve then the corticosteroids should be tapered over four weeks. In addition, the physician should continue to monitor LFT’s every 2-3 days unless the patient is stable. Mycophenolate mofetil (500 mg every 12 hours) can be added in cases refractory to corticosteroids. Infliximab should not be given to hepatitis patients as it carries the risk of hepatotoxicity.

## Conclusion

In conclusion, immunotherapeutic checkpoint inhibitors have revolutionized the management of numerous solid tumors by clearly displaying improved mortality benefit. As many more monoclonal antibodies are expected to become available in the near future, the need to understand their side effect profile has become imperative. Oncologists, pathologists, and gastroenterologists in particular, should remain vigilant to the gastrointestinal and liver related adverse effects cancer immunotherapy causes. In addition, the management of such sequelae should be acknowledged by clinicians across all multi-disciplinary fields of medicine. Many more large studies are needed to determine exactly which immunotherapeutic agents are more or less likely to cause gastrointestinal and liver adverse effects. Moreover, the need to stratify for theses adverse effects between various at risk subsets of patients undergoing cancer treatment with new checkpoint inhibitors—remains an important study for the future.

## Conflict of interests

The authors declare that they have no conflict of interest.

## References

[1] Administration, USFDA (2017). FDA approved drug products.

[2] Cameron F, Whiteside G, Perry C (2011). Ipilimumab: first global approval. Drugs.

[3] Wang DY, Salem JE, Cohen JV, Chandra S, Menzer C, Ye F (2018). Fatal Toxic Effects Associated With Immune Checkpoint Inhibitors: A Systematic Review and Meta-analysis. JAMA Oncol.

[4] Puzanov I, Diab A, Abdallah K, Bingham CO 3rd, Brogdon C, Dadu R (2017). Managing toxicities associated with immune checkpoint inhibitors: consensus recommendations from the Society for Immunotherapy of Cancer (SITC) Toxicity Management Working Group. J Immunother Cancer.

[5] Hodi FS, O'Day SJ, McDermott DF, Weber RW, Sosman JA, Haanen JB (2010). Improved survival with ipilimumab in patients with metastatic melanoma. N Engl J Med,..

[6] Wolchok JD, Neyns B, Linette G, Negrier S, Lutzky J, Thomas L (2010). Ipilimumab monotherapy in patients with pretreated advanced melanoma: a randomised, double-blind, multicentre, phase 2, dose-ranging study. Lancet Oncol.

[7] Klair JS, Girotra M, Hutchins LF, Caradine KD, Aduli F, Garcia-Saenz-de-Sicilia M (2016). Ipilimumab-induced gastrointestinal toxicities: a management algorithm. Dig Dis Sci.

[8] Gonzalez RS, Salaria SN, Bohannon CD, Huber AR, Feely MM, Shi C (2017). PD-1 inhibitor gastroenterocolitis: case series and appraisal of 'immunomodulatory gastroenterocolitis'. Histopathology.

[9] Zhou W, Huang Y, Lai J, Lu J, Feely M, Liu X (2018). Anti-Inflammatory Biologics and Anti-Tumoral Immune Therapies-Associated Colitis: A Focused Review of Literature. Gastroenterol Res.

[10] Marthey L, Mateus C, Mussini C, Nachury M, Nancey S, Grange F ( 2016). Cancer Immunotherapy with anti-CTLA-4 monoclonal antibodies induces an inflammatory bowel disease. J Crohns Colitis.

[11] Pagès C, Gornet JM, Monsel G, Allez M, Bertheau P, Bagot M (2013). Ipilimumab-induced acute severe colitis treated by infliximab. Melanoma Res.

[12] Minor DR, Chin K, Kashani-Sabet M (2009). Infliximab in the treatment of anti-CTLA4 antibody (ipilimumab) induced immune-related colitis. Cancer Biother Radiopharm.

[13] Merrill SP, Reynolds P, Kalra A, Biehl J, Vandivier RW, Mueller SW (2014). Early administration of infliximab for severe ipilimumab-related diarrhea in a critically ill patient. Ann Pharmacother.

[14] Johnson DH, Zobniw CM, Trinh VA, Ma J, Bassett RL Jr, Abdel-Wahab N (2018). Infliximab associated with faster symptom resolution compared with corticosteroids alone for the management of immune-related enterocolitis. J Immunother Cancer.

[15] Mir R, Shaw HM, Nathan PD (2019). Mycophenolate mofetil alongside high-dose corticosteroids: optimizing the management of combination immune checkpoint inhibitor-induced colitis. Melanoma Res.

[16] Abu-Sbeih H, Ali FS, Alsaadi D, Jennings J, Luo W, Gong Z, Richards DM (2018). Outcomes of vedolizumab therapy in patients with immune checkpoint inhibitor-induced colitis: a multi-center study. J Immunother Cancer.

[17] Wang Y, Wiesnoski DH, Helmink BA, Gopalakrishnan V, Choi K, DuPont HL (2018). Fecal microbiota transplantation for refractory immune checkpoint inhibitor-associated colitis. Nat Med.

[18] Weber J, Thompson JA, Hamid O, Minor D, Amin A, Ron I, Ridolfi R (2009). A randomized, double-blind, placebo-controlled, phase II study comparing the tolerability and efficacy of ipilimumab administered with or without prophylactic budesonide in patients with unresectable stage III or IV melanoma. Clin Cancer Res.

[19] Weber JS, Kähler KC, Hauschild A (2012). Management of immune-related adverse events and kinetics of response with ipilimumab. J Clin Oncol.

[20] Ribas A, Kefford R, Marshall MA, Punt CJ, Haanen JB, Marmol M (2013). Phase III randomized clinical trial comparing tremelimumab with standard-of-care chemotherapy in patients with advanced melanoma. J Clin Oncol.

[21] Bernardo SG, Moskalenko M, Pan M, Shah S, Sidhu HK, Sicular S (2013). Elevated rates of transaminitis during ipilimumab therapy for metastatic melanoma. Melanoma Res.

[22] Hammers HJ, Plimack ER, Infante JR, Rini BI, McDermott DF, Lewis LD (2014). Phase I study of nivolumab in combination with ipilimumab in metastatic renal cell carcinoma (mRCC). J Clin Oncol.

[23] OPDIVO U.S Prescribing Information.

[24] Hamid O, Robert C, Daud A, Hodi FS, Hwu WJ, Kefford R et al (2013). Safety and tumor responses with lambrolizumab (anti-PD-1) in melanoma. N Engl J Med.

[25] Robert C, Thomas L, Bondarenko I, O'Day S, Weber J, Garbe C (2011). Ipilimumab plus dacarbazine for previously untreated metastatic melanoma. N Engl J Med.

[26] Kim KW, Ramaiya NH, Krajewski KM, Jagannathan JP, Tirumani SH, Srivastava A (2013). Ipilimumab associated hepatitis: imaging and clinicopathologic findings. Invest New Drugs.

[27] Ribas A, Hodi FS, Callahan M, Konto C, Wolchok J (2013). Hepatotoxicity with combination of vemurafenib and ipilimumab. N Engl J Med.

[28] Important Safety Information about YERVOY® (ipilimumab).

